# Neuronal Injury Model Divulges Differences in Dendrite and Axonal Function and Regeneration in Adults

**DOI:** 10.1523/ENEURO.0207-24.2024

**Published:** 2024-08-23

**Authors:** Lauren S. Vaughn, Jinyoung Lee

**Affiliations:** University of South Carolina, Columbia, South Carolina 29208

While injury responses are generally well studied, the focus tends to be on axon regeneration, leaving dendrite-specific contribution after injury and the regeneration process less understood. After axons are injured, the area distal to the crush site degenerates, and injury signaling is initiated by a retrograde calcium wave that activates stress signaling including dual leucine zipper kinase (DLK)/JNK activation, transcription of regeneration associated genes, and directed local translation which allows ultimately for reinitiation of axon outgrowth. Potential for outgrowth is greater in the peripheral nervous system as compared with that in the central. Dendrites, like axons, are vulnerable to injury from stroke, seizure, and traumatic injury, but limitations in the ability to generate a targeted injury and the inability to track dendrites of individual cells in vertebrate models make the study of dendrite injury and regeneration difficult. Advancements have been made in the use of lasers to induce precise injury, allowing studies to be conducted in invertebrates (flies, worms) and vertebrates (zebrafish), though both adult neuron and mammalian studies have still been limited. Dendrites and axons have well-established differences in their ability to locally respond to external stimuli, so it stands that the intrinsic ability and mechanism of regeneration may also differ.

Dendrites and axons are typically classified by canonical differences in function and anatomy/structural properties, including differential composition and localization of organelles and cytoskeleton, trafficking and localization of proteins and mRNA, and local translation (Rangaraju et al., 2017; Goaillard et al., 2019). These classical characterizations do not always accurately reflect the diversity of structures as well as functions that can be carried out by dendrites and axons, which may vary widely by cell type. [These “misbehaving” neurons are nicely reviewed in Goaillard et al. (2019).] Generally, neuronal function is thought of as a typical flow of information where dendrites passively receive information that is transferred and processed through the soma to the axon, an action potential is propagated to the axon terminal, and the message is relayed to the next cell though the actual neuronal landscape is much more complex. Variations from the dogma include neurons that only have populations of dendrites or axons, dendrites that can propagate action potentials and release neurotransmitters, and axons that form synapses with other axons. As the roles of both dendrites and axons are complex and essential for proper neuronal function, it stands that proper regeneration after injury of both is essential to restore full functionality.

Previous studies have been able to show that both axons and dendrites are capable of regeneration following injury in flies and worms. There is additional evidence that dendrites have some regenerative capacity in both the central and peripheral nervous systems of zebrafish and mouse injury models, though it is unclear how dendrite damage is sensed. Additionally, it is unclear how well dendrites of mature neurons regenerate in the absence of the remodeling found during development. Interestingly, dendritic regeneration has been found to be independent of DLK, which is essential for axon regeneration (Stone et al., 2014; Brar et al., 2022). This highlights the importance of deciphering the molecular mechanisms of both axon and dendrite regeneration to understand how injured neurons are fully reconstructed to attempt to achieve functional restoration in adult neurons.

**Figure 1.**
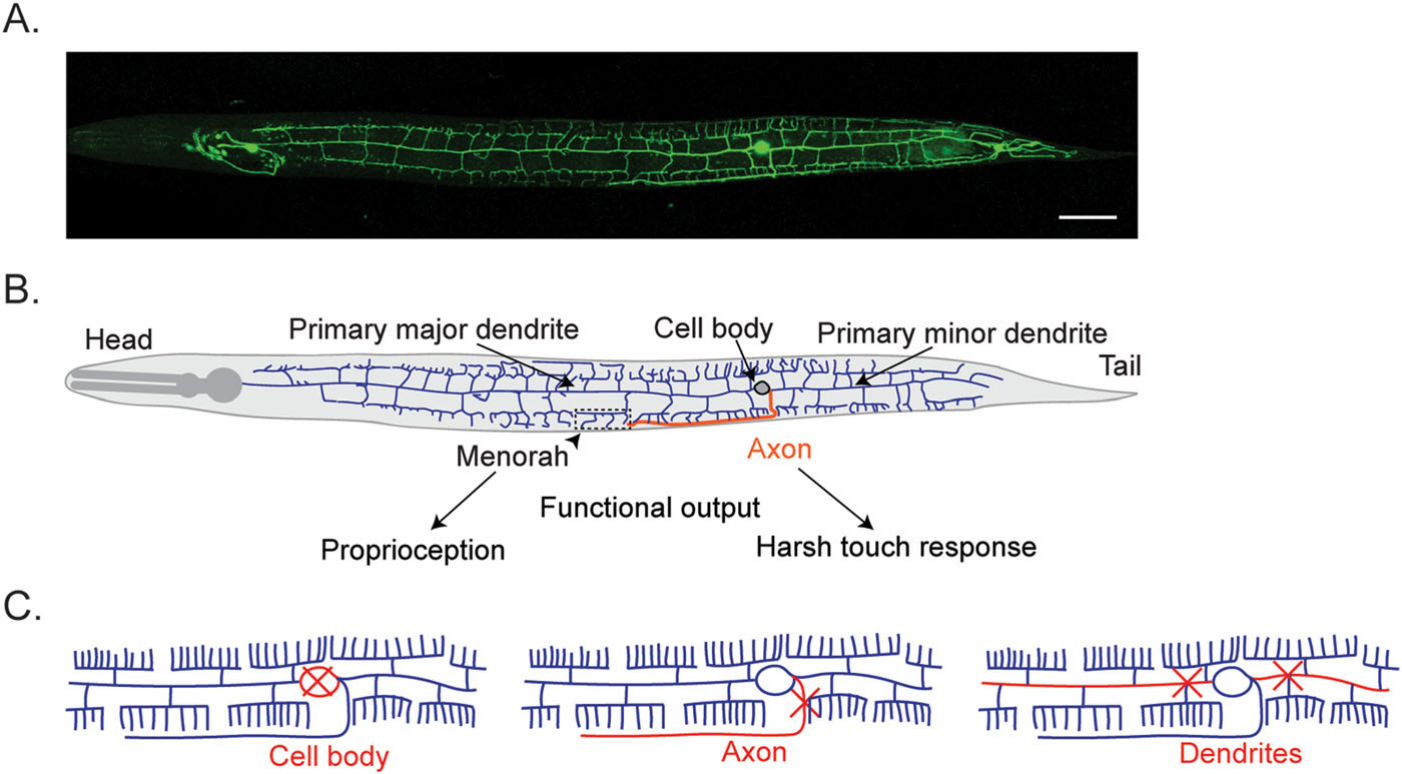
PVD neuron and injury model. ***A***, Confocal image of PVD neuron expressing wdls52 (pF49H12.4::GFP); scale bar, 20 micron. ***B***, PVD neuron schematic of dendrites (blue), soma (gray), and axon (orange). ***C***, Location of PVD neuron injury sites for each target (Brar et al., 2024).

Due to their distinct dendritic structures, recent studies have used dendritic arborization (da) neurons in *Drosophila* larvae and PVD neurons in *Caenorhabditis elegans* (*C. elegans*) as in vivo model systems to demonstrate dendritic regrowth after injury. Work in da neurons began to establish the link between specific behaviors and intact dendrites specifically (Thompson-Peer et al., 2016). PVD neurons of *C. elegans* are highly polarized neurons with well-defined axonal and dendritic compartments capable of responding to a wide range of external stimuli (Fig. 1*A*,*B*). Like what was found on da neurons, recent studies found that the dendrites and axons of PVD neurons may independently regulate proprioception and harsh touch sensation behaviors (Tao et al., 2019).

The recent eNeuro article by Brar et al. (2024) established an injury model of PVD neurons, whereby distinct roles of dendrites and axons functional behavior can be characterized. By comparing ablated, axotomized, and dendrotomized PVD neurons, they established that both ablation and axonal injury affect nociceptive harsh touch responses (Fig. 1*C*). After ablation or axotomy, the number of worms capable of responding to harsh touch stimuli was significantly decreased as well as the locomotion after stimulus. This role is specific to axons as harsh touch was unaffected even when both sides were dendrotomized or if dendrite-defective mutants were used.

This injury model also allowed for the investigation into whether axon regeneration led to functional recovery. After injury, the canonical retraction of the axon at the injury site was observed followed by new neurite formation (6 h postinjury) and reconnection with its distal counterpart (24 h postinjury). Regeneration was accompanied by functional recovery, as 24 h after injury the number of worms responding to harsh touch was not significantly different from the mock injury worms. This regeneration was shown to be dependent on DLK-1/MLK-1 signaling in concordance with other axonal regeneration studies.

Previous work indicated that proprioception and harsh touch sensation behaviors may by independently regulated (Tao et al., 2019). Brar et al. (2024) tested if proprioception behavior was changed after injury by analyzing aspects of the sinusoidal trajectory of the moving worms. After injury of the major primary dendrite, amplitude and bending angle were significantly decreased, while wavelength was only significantly decreased after injury to both the major and minor primary dendrite when the injured PVD was in close contact with the plate. Notably, none of the measured postures were altered in axotomized worms supporting the idea that axons and dendrites of PVD neurons are independently and distinctly regulating behaviors.

Similar to the regeneration seen after axotomy, 24 h after dendrotomy, a significant increase in all measured postures was observed. Functional recovery in posture behavior correlated with the type of dendrite regeneration events, particularly menorah–menorah fusion and reconnection between proximal and distal branches of the injured dendrite. Dendrite regeneration was found to be independent of DLK-1/MLK-1 signaling, unlike axon regeneration (Brar et al., 2024).

Overall, the findings from Brar et al. (2024) indicate that PVD neuron axons and dendrites regulate nociception and proprioception, respectively, in worms. Furthermore, dendrite and axon regeneration are essential for the restoration of these sensory modalities. The distinct functions and regenerative mechanisms further demonstrate the need for more in-depth studies into how injuries are sensed in dendrites and regeneration initiated, especially in established adult neurons.

Due to the challenge of both specific injury and postinjury monitoring of dendrites in mammals, labs have shifted their focus to *Drosophila* and *C. elegans* laser ablation/-otomy models. While laser ablation does not fully recapitulate a classical dendrite or axon injury in vivo, current tools do not allow for specific targeted injury of dendrites in the CNS of vertebrates without damaging surrounding tissues, complicating outcomes and interpretation of distinct roles within neuronal structure. The PVD injury model utilized in Brar et al. (2024) allows for the ability to pick apart differences in regeneration as well as functional input and output of dendrites and axons, though future studies will be needed to uncover the molecular signaling that mediates response and regeneration. It remains unclear how well the molecular mechanisms identified in invertebrates will extrapolate to vertebrate models or more complex models of traumatic injury.

Ultimately, the model used in Brar et al. (2024) is elegant in its simplicity, adding to the “classical” model of a neuron but highlighting that there is much more beneath the surface when it comes to the distinct function and structure of dendrites. This work demonstrates that regeneration after injury of both dendrites and axons are likely necessary for full functional recovery of a neuron. This model of injury and regeneration has helped to not only establish differential roles of dendrites and axons but also highlight that dendrite regeneration cannot simply be treated as the younger sibling of axon regeneration.
